# Multimodal ultrasound fusion network for differentiating between benign and malignant solid renal tumors

**DOI:** 10.3389/fmolb.2022.982703

**Published:** 2022-09-06

**Authors:** Dongmei Zhu, Junyu Li, Yan Li, Ji Wu, Lin Zhu, Jian Li, Zimo Wang, Jinfeng Xu, Fajin Dong, Jun Cheng

**Affiliations:** ^1^ Department of Ultrasound, The Second Clinical Medical College, Jinan University, Shenzhen, China; ^2^ Department of Ultrasound, The Affiliated Nanchong Central Hospital of North Sichuan Medical College, Nanchong, China; ^3^ National-Regional Key Technology Engineering Laboratory for Medical Ultrasound, School of Biomedical Engineering, Health Science Center, Shenzhen University, Shenzhen, China; ^4^ Medical Ultrasound Image Computing (MUSIC) Laboratory, Shenzhen University, Shenzhen, China; ^5^ Marshall Laboratory of Biomedical Engineering, Shenzhen University, Shenzhen, China; ^6^ Department of Urology Surgery, The Affiliated Nanchong Central Hospital of North Sichuan Medical College, Nanchong, China

**Keywords:** renal tumor, artificial intelligence, classification, deep learning, contrast-enhanced ultrasound

## Abstract

**Objective:** We aim to establish a deep learning model called multimodal ultrasound fusion network (MUF-Net) based on gray-scale and contrast-enhanced ultrasound (CEUS) images for classifying benign and malignant solid renal tumors automatically and to compare the model’s performance with the assessments by radiologists with different levels of experience.

**Methods:** A retrospective study included the CEUS videos of 181 patients with solid renal tumors (81 benign and 100 malignant tumors) from June 2012 to June 2021. A total of 9794 B-mode and CEUS-mode images were cropped from the CEUS videos. The MUF-Net was proposed to combine gray-scale and CEUS images to differentiate benign and malignant solid renal tumors. In this network, two independent branches were designed to extract features from each of the two modalities, and the features were fused using adaptive weights. Finally, the network output a classification score based on the fused features. The model’s performance was evaluated using five-fold cross-validation and compared with the assessments of the two groups of radiologists with different levels of experience.

**Results:** For the discrimination between benign and malignant solid renal tumors, the junior radiologist group, senior radiologist group, and MUF-Net achieved accuracy of 70.6%, 75.7%, and 80.0%, sensitivity of 89.3%, 95.9%, and 80.4%, specificity of 58.7%, 62.9%, and 79.1%, and area under the receiver operating characteristic curve of 0.740 (95% confidence internal (CI): 0.70–0.75), 0.794 (95% CI: 0.72–0.83), and 0.877 (95% CI: 0.83–0.93), respectively.

**Conclusion:** The MUF-Net model can accurately classify benign and malignant solid renal tumors and achieve better performance than senior radiologists.

**Key points:** The CEUS video data contain the entire tumor microcirculation perfusion characteristics. The proposed MUF-Net based on B-mode and CEUS-mode images can accurately distinguish between benign and malignant solid renal tumors with an area under the receiver operating characteristic curve of 0.877, which surpasses senior radiologists’ assessments by a large margin.

## Introduction

Nowadays, cancer remains a serious threat to human health worldwide. The incidence of renal cancer is increasing annually, with more than 400,000 new cases every year worldwide (Shingarev and Jaimes, 2017; Sung et al., 2021). Clear cell renal cell carcinoma (ccRCC) is the most common type of renal cell carcinoma (RCC), accounting for 80% of all RCCs. Most renal tumors do not cause obvious clinical symptoms (Ljungberg et al., 2019). About 20%∼30% of patients with renal tumor resection were preoperatively misdiagnosed, resulting in unnecessary surgery with a final post-surgical diagnosis of being benign (Schachter et al., 2007). The diagnostic accuracy needs to be improved, especially for differentiating between hypoechoic benign solid tumors and malignant tumors. Noninvasive imaging modalities such as ultrasound, computed tomography (CT), and magnetic resonance imaging (MRI) have improved sensitivity and specificity in preoperatively differentiating among benign, malignant, and borderline tumors. Compared with these imaging modalities, contrast-enhanced ultrasound (CEUS) is more sensitive in visualizing the microcirculatory perfusion characteristics of renal tumors and thus is widely used. However, the diagnostic accuracy varies in terms of different lesion locations and radiologists.

Deep learning has shown promising results in the classification and diagnosis of renal tumors over the past few years (Oktay et al., 2018; Hussain et al., 2021; Wang et al., 2021), which does not require subjectively defined features and can capture the entirety of biological information from images compared with traditional machine learning (Sun et al., 2020; Bhandari et al., 2021; Giulietti et al., 2021; Khodabakhshi et al., 2021). The literature indicates that deep learning algorithms are better than human experts in diagnosing many kinds of diseases, such as liver, breast, lung, fundus, skin lesions (Wu et al., 2017; Lin et al., 2020; Li et al., 2021; Liu et al., 2021). These studies have shown that deep learning is stable and generalizable and can compensate for the diagnostic discrepancy among doctors with different levels of experience. To the best of our knowledge, there are no ultrasound-based radiomics studies for the differentiation between benign and malignant solid renal tumors (Esteva et al., 2017; Kokil and Sudharson, 2019; Zabihollahy et al., 2020; Hu et al., 2021; Mi et al., 2021).

In this study, we aim to establish a multimodal fusion deep neural network based on gray-scale ultrasound and CEUS images to discriminate between benign and malignant solid renal tumors. The performance of the multimodal fusion model is compared with that of the models built on single-modal data, as well as junior and senior radiologists’ assessments.

## Materials and methods

### Patients

This retrospective study was approved by our joint institutional review boards, and anonymized data was shared through a data-sharing agreement between institutions (The Second Clinical Medical College, Jinan University, and The Affiliated Nanchong Central Hospital of North Sichuan Medical College) (No. 18PJ149 and No. 20SXQT0140). Individual consent for this retrospective analysis was waived. From June 2012 to June 2021, the information for 1547 cases was obtained from the surgical pathology database in the Pathology Department of The Second Clinical Medical College of Jinan University and The Affiliated Nanchong Central Hospital of North Sichuan Medical College.

**FIGURE 1:**
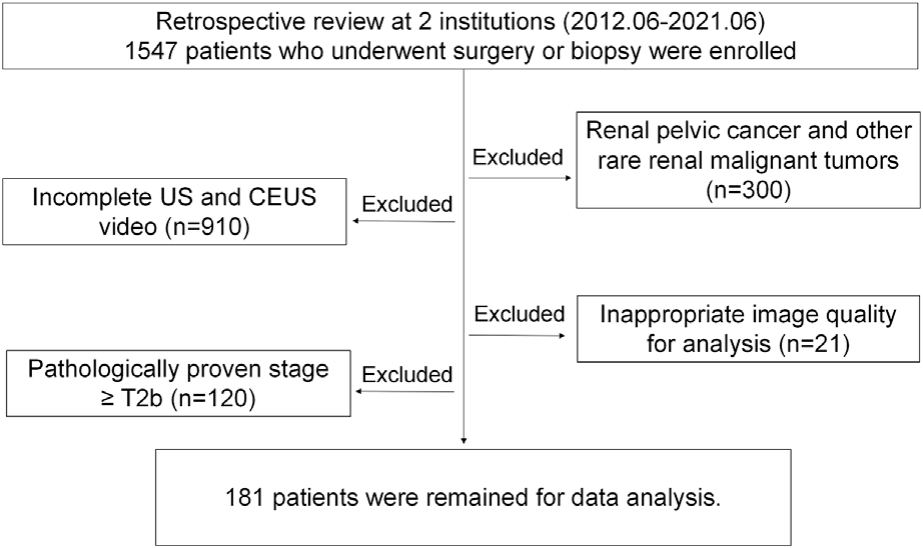
Flow diagram of patient enrollment.

The inclusion criteria were as follows: (1) preoperative CEUS examinations were performed before surgery, and (2) all cases were confirmed by pathological diagnosis after surgery. Patients were excluded based on the following criteria: (1) renal pelvis cancer and other rare types of renal malignancies; (2) patients did not receive the ultrasound and CEUS examinations; (3) poor image quality, and (4) pathologic stage ≥ T2b. Figure 1 shows the flow diagram of patient enrollment for this study. Finally, 181 patients (100 solid malignant tumors and 81 solid benign tumors) were left. Patients’ demographic and clinical characteristics are shown in Table 1.

**TABLE 1:** Patient characteristics.

	Malignant (*n* = 100)	Benign (*n* = 81)	*p* Value
Gender: n (%)			< 0.001*
Male	74 (74.0%)	23 (28.4%)	
Female	26 (26.0%)	58 (71.6%)	
Age: mean ± STD	58.36 ± 14.06	53.31 ± 14.00	0.017*
BMI: mean (IQR)	23.0 (22.0–25.0)	23.0 (21.0–24.0)	0.275
Tumor mean size: mean (IQR)	4.0 (3.0–6.0)	4.0 (3.0–5.0)	0.918
Clinical sign: n (%)			0.588
Waist discomfort/Fatigue	46 (46.0%)	34 (42.0%)	
No symptoms	54 (54.0%)	47 (58.0%)	
Surgery: n (%)			0.475
Partial nephrectomy	41 (41.0%)	29 (35.8%)	
Radical nephrectomy	59 (59.0%)	52 (64.2%)	

BMI, body mass index; IQR, interquartile range; STD, standard deviation.

*Statistically significant.

### Contrast-enhanced ultrasound Imaging

CEUS examinations were performed using the following three ultrasound systems: LOGIQ E9 (GE Healthcare, Unites States), Resona7 (Mindray Ultrasound Systems, China), and IU22 (Philips Medical Systems, Netherlands), with a 1.0–5.0 MHz convex probe. CEUS was carried out by ultrasound machines with contrast-specific software and a bolus of 1.0∼1.2 ml microbubble contrast agent (SonoVue; Bracco, Milan, Italy) via an antecubital vein followed by 5.0 ml normal saline with a peripheral 18∼22 G needle. The CEUS digital video was at least 3∼5 min long each time. During contrast-enhanced imaging, low-acoustic power modes were used with a mechanical index of 0.05∼0.11. All the CEUS examination videos were retrospectively analyzed by two groups of radiologists with different levels of experience (three junior radiologists with more than 5∼6 years of experience in CEUS imaging and three senior radiologists with more than 10∼15 years of experience in CEUS imaging).

In this study, we used the following phase terms: (1) cortical phase began 10∼15 s after injection, and (2) medullary phase approximately began 30∼45 s after injection until the microbubble echoes disappeared. The entire course of CEUS was saved as Digital Imaging and Communication in Medicine format.

### Data annotation and preprocessing

**FIGURE 2:**
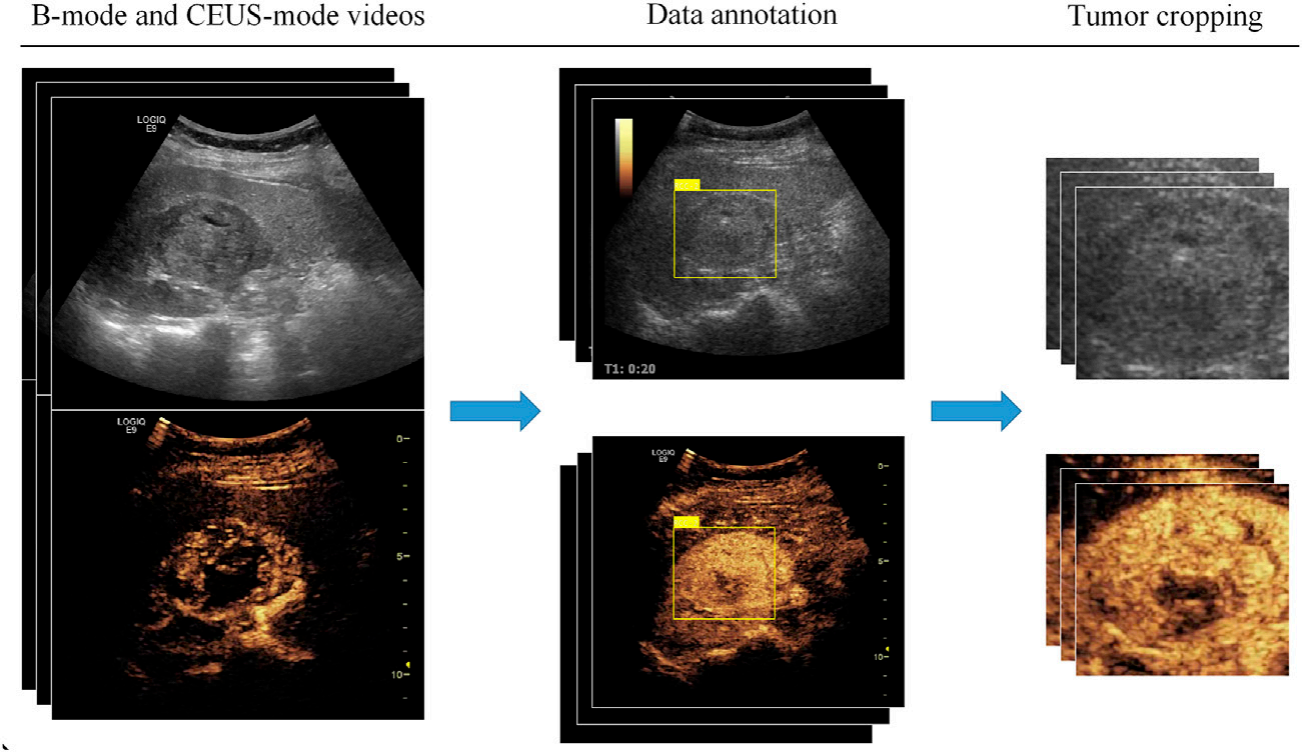
Data annotation and preprocessing.

CEUS videos were annotated using the Pair annotation software package (Liang et al., 2022; Qian et al., 2022). In each CEUS video, about 50∼60 images were selected from the cortical and medullary phases. A senior radiologist classified the tumor as either benign or malignant and annotated its location in each selected image by a bounding box. Then, according to the bounding boxes, these images were cropped into smaller images as region of interest to exclude the non-tumor regions (Figure 2). This resulted in a total of 9794 images, of which 3659 images were benign (including 1531 from 36 atypical benign cases and 2128 images from 45 typical benign cases and 6135 images were malignant (including 2964 images from 62 ccRCC cases; 2114 images from 25 pRCC cases; 1057 images from 13 chRCC cases) (Table 2).

**TABLE 2:** Number distribution of patients and images among histologic types.

	Benign			Malignant			
	Total	Atypical	Typical	Total	ccRCC	pRCC	chRCC
Patients	81	36	45	100	62	25	13
Images	3659	1531	2128	6135	2964	2114	1057

ccRCC, clear cell Renal cell carcinoma; chRCC, chromophobe renal cell carcinomas; pRCC, papillary renal cell carcinomas.

### Multimodal ultrasound fusion network

The dataset used in this study contained B-mode and CEUS-mode images, and they were in one-to-one correspondence. Therefore, we proposed the MUF-Net to take full advantage of the multimodal features, which can independently extract features from each of the two modalities and learn adaptive weights to fuse features for each sample.

**FIGURE 3:**
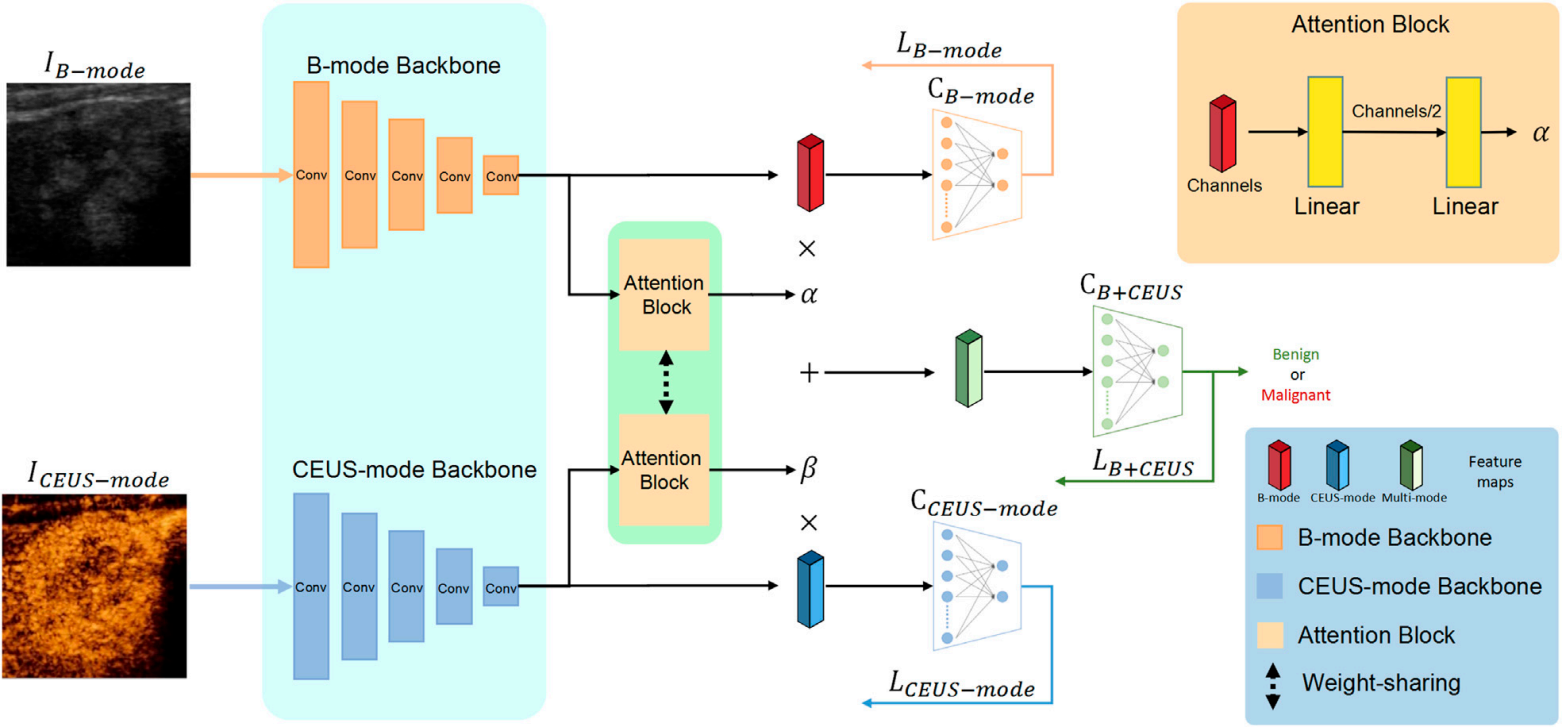
Overall architecture of the proposed MUF-Net framework.

The overall architecture of MUF-Net is shown in Figure 3, $I_{B-mode}$ and $I_{CEUS-mode}$ represented the B-mode and CEUS-mode images, respectively. We used two independent EffecientNet-b3 as the backbone to extract features from B-mode and CEUS-mode images. The backbone had an input size of 300 × 300 × 3 and an output size of 10 × 10 × 1536 after five down sampling blocks. To reduce the parameters of the network and prevent overfitting, we used a global average pooling layer to downsample the output feature maps of each backbone from 10 × 10 × 1536 to 1 × 1 × 1536. Subsequently, we fused the features of the two modalities. Considering that the features of the two modalities in each sample may contribute differently to the final prediction, we designed two attention blocks sharing weights to produce adaptive weights α and β for modality fusion. The feature maps of the two modalities were subsequently weighted and summed based on the adaptive weights, yielding a fused feature map of 1 × 1 × 1536. Finally, through a fully connected layer and a softmax layer, the classification result was given.

Notably, to improve the feature learning ability of each backbone, we added two classifiers $C_{B-mode}$ and $C_{CEUS-mode}$ for each single modality, as shown in Figure 3, which independently took B-mode features and CEUS-mode features as input and calculated the loss of each mode ( $L_{B-mode}\;$ and $L_{CEUS-mode}$ ), respectively. The total loss was defined by Eq. 1. The two losses, $L_{B-mode}$ and $L_{CEUS-mode}$ , were only used during training, and the final classification result was given by the multimodal classifier, $C_{B+CEUS}$ . Due to this reason, the multimodal loss, $L_{B+CEUS}$ , had a higher weight than the other two losses. For the calculation of the three losses in Eq. 1, we employed the class-balanced focal loss (Zhang et al., 2021).

$$L_{total}=3L_{B+CEUS}+L_{B-mode}+L_{CEUS-mode}$$ (1)

### Implementation details

All experiments were conducted using five-fold cross-validation. For data splitting, we ensured that the images from the same patient went into either the training set or the test set to avoid the data leakage problem. To avoid model overfitting, data augmentation techniques were applied to the training set, which included random spatial transformations, random non-rigid body transformations, and random noise.

The dataset used in this study had a weak class imbalance problem. The ratio of benign images to malignant images was 3:5. Re-sampling techniques (Zhang et al., 2021) were popularly used for dealing with long-tailed problems. We used class-balanced sampling to alleviate class imbalance by first sampling a class and then selecting an instance from the chosen class (Kang et al., 2019).

All backbones used in these experiments were pretrained on ImageNet. All models used in the experiments were implemented by PyTorch on a NVIDIA 3090 GPU. The stochastic gradient descent optimizer was used with a learning rate of 0.05 which was halved every 10 epochs. In each round of five-fold cross-validation, models based on B-mode, CEUS-mode, and B + CEUS mode were trained for 100 epochs, respectively, and the models with the highest accuracy on the validation set were saved.

### Radiologists’ assessments

Original uncropped CEUS videos and images were evaluated by three junior and three senior radiologists and manually classified as benign or malignant. The radiologists were blinded to any clinical information of the patients. Intraclass correlation coefficients (ICCs) were used to evaluate the inter-rater agreement within each radiologist group, with an ICC greater than 0.75 indicating good agreement.

### Statistical analysis

All statistical analyses were performed using the SciPy package in Python (version 3.8). Depending on whether data conformed to a normal distribution, continuous variables were compared using the Student’s t-test or the Mann-Whitney U test. The non-ordered categorical variables were compared by the chi-square test. Receiver operating characteristic (ROC) curve analysis was used to evaluate the performance of junior radiologists, senior radiologists, individual modality-based networks, and MUF-Net. In addition, we also used other metrics to evaluate model performance from various aspects, including sensitivity, specificity, positive predictive value (PPV), and negative predictive value (NPV). Comparison of the difference between areas under the ROC curve (AUCs) was performed using the Delong test. A two-sided *p* value < 0.05 was considered statistically significant.

## Results

### Patient characteristics

The age of the patients in the benign tumor group was less than that of the patients in the malignant tumor group (58.36 ± 14.06 vs. 53.31 ± 14.00 years) (*p* = 0.017). Regarding gender distribution, there was a significant difference between these two groups (*p* considered statistically <0.001), with male patients being more frequent in the malignant group than in the benign group. Patient characteristics are shown in Table 1.

### Performance of radiologists’ assessments

**FIGURE 4:**
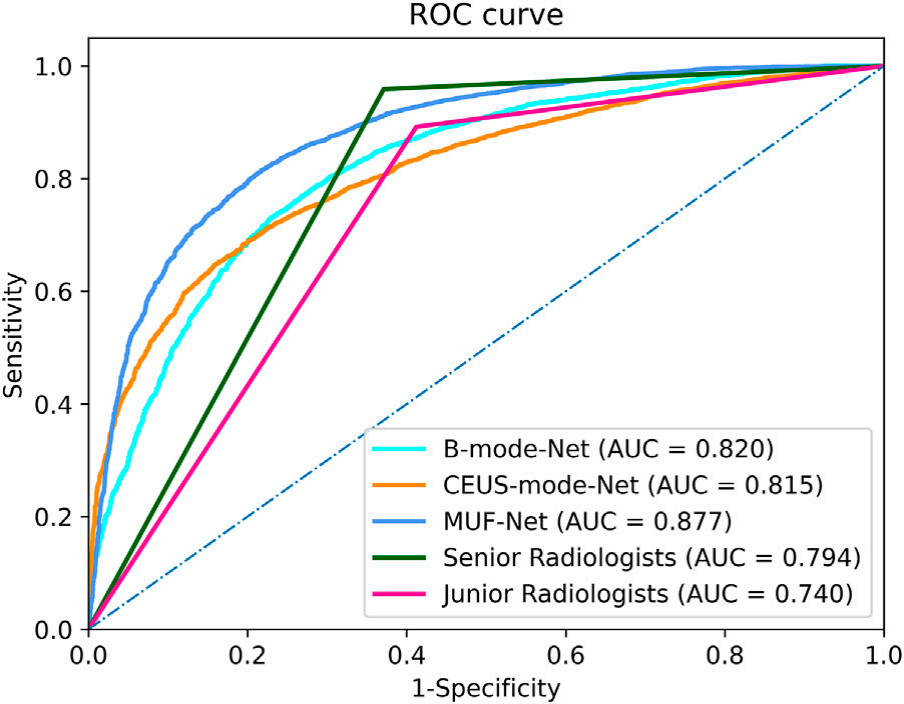
The receiver operating characteristic curves of the MUF-Net, single-mode models, and radiologists’ assessments in the test cohort.

The ICCs in the junior and senior radiologist groups were 0.81 and 0.83, respectively, indicating good inter-rater agreement. Each radiologist classified a tumor as benign or malignant, and the radiologists’ assessments in each group were merged by majority voting. The performance of radiologists’ assessments is shown in Table 3. The AUC, accuracy, sensitivity, specificity, PPV, and NPV of junior radiologists were 0.740 (95% confidence interval (CI): 0.70–0.75), 70.6%, 89.3%, 58.7%, 58.0%, and 89.5%, respectively. The AUC, accuracy, sensitivity, specificity, PPV, and NPV of senior radiologists were 0.794 (95% CI: 0.72–0.83), 75.7%, 95.9%, 62.9%, 62.3%, and 95.9%, respectively. The ROC curves for the test set were shown in Figure 4.

**TABLE 3:** Classification performance of deep learning models and radiologists.

	AUC (95% CI)	Accuracy (%)	Sensitivity (%	Specificity (%)	PPV (%)	NPV (%)
Junior radiologists	0.740 (0.70–0.75)	70.6	89.3	58.7	58.0	89.5
Senior radiologists	0.794 (0.72–0.83)	75.7	95.9	62.9	62.3	95.9
B-mode-Net	0.820 (0.70–0.83)	74.5	75.0	77.0	73.4	62.3
CEUS-mode-Net	0.815 (0.75–0.89)	73.9	73.8	73.2	72.5	62.2
MUF-Net	0.877 (0.83–0.93)	80.0	80.4	79.1	86.9	70.0

CI, confidence interval; CEUS-mode, contrast-enhanced ultrasound mode; MUF-Net, multimodal ultrasound fusion network; AUC, area under the receiver operating characteristic curve; PPV, positive predictive value; NPV, negative predictive value.

### Performance of deep learning models

The AUC, accuracy, sensitivity, specificity, PPV, and NPV of EffecientNet-b3 network trained on B-mode images were 0.820 (95% CI: 0.72–0.83), 74.5%, 75.0%, 77.0%, 73.4%, and 62.3%, respectively (Table 3). The AUC, accuracy, sensitivity, specificity, PPV, and NPV of EffecientNet-b3 network trained on CEUS-mode images were 0.815 (95% CI: 0.75–0.89), 73.9%, 73.8%, 73.2%, 72.5%, and 62.2%, respectively. The AUC, accuracy, sensitivity, specificity, PPV, and NPV of MUF-Net trained on B-mode and CEUS-mode images were 0.877 (95% CI: 0.83–0.93), 80.0%, 80.4%, 79.1%, 86.9%, and 70.0%, respectively. The proposed MUF-Net significantly outperformed junior and senior radiologists (*p* < 0.001).

## Discussion

This study explored the performance of deep learning based on ultrasound images for benign/malignant classification of solid renal tumors. We proposed the MUF-Net for fusing complementary features of two modalities, which used two independent EffecientNet-b3 as backbones to extract features from B-mode and CEUS-mode ultrasound images. Our method reached expert-level diagnostic performance and had a higher diagnostic PPV compared with radiologists.

**FIGURE 5:**
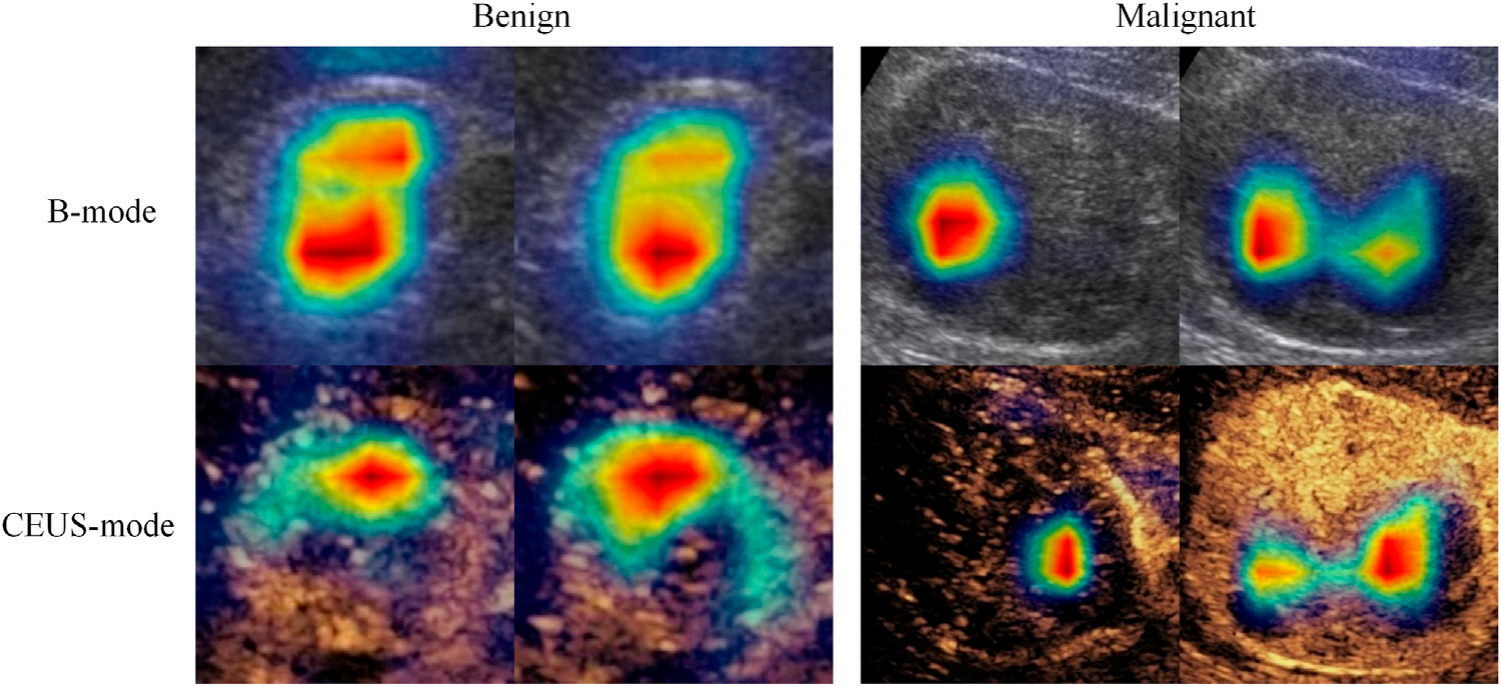
Feature heatmaps of a benign tumor and a malignant tumor to show B-mode and CEUS-mode images contain complementary information for diagnosis. The red color represents higher weights (i.e., the network pays more attention to this region).

CEUS is an important supplement to conventional ultrasound, CT, and MRI in diagnosing solid renal tumors. Compared with conventional ultrasound, CEUS can display perfusion characteristics of renal tumors in cortical and medullary phases in real-time, which is an important tool for improving differential diagnosis of benign and malignant renal tumors. In this study, we observed that the deep learning models built on either B-mode or CEUS-mode images achieved better performance than junior or senior radiologists. Moreover, the deep learning model combing B-mode and CEUS-mode images further improved the classification performance. This indicates that B-mode and CEUS-mode images have complementary information for diagnosing solid renal tumors. To verify this point, we used class activation maps (Xu et al., 2022) to visualize the important regions that the model paid attention to in B-mode and CEUS-mode images. As shown in Figure 5, the important regions contributing to the final prediction were different between B-mode and CEUS-mode images. In other words, the MUF-Net can automatically extract complementary features from different modalities to improve the classification performance.

According to the results from the comparative experiments, we found that the performance was similar between the two models built on B-mode or CEUS-mode images. The B-mode images-based model had slightly better performance, which might be due to the different microcirculatory perfusion characteristics of solid renal tumors. Solid renal tumors of different histopathological types have different vascular density, fat content, blood flow velocity, and the severity of arteriovenous fistulas.

Lin et al. reported an AUC of 0.846 for the classification of benign and malignant renal tumors on enhanced CT images using inception-v3 (Lin et al., 2020). Xu et al. used ResNet-18 to classify multimodal MRI images of renal tumors, with AUCs of 0.906 and 0.846 on T2WI and DWI, respectively (Xu et al., 2022). The AUC was improved to 0.925 by fusing the two modalities, exceeding the diagnostic performance of highly qualified radiologists. The results of this study were similar. The MUF-Net based on multimodal data surpassed the models based on individual modalities by a large margin. Therefore, we inferred that the adaptive weights learned by MUF-Net could help the network acquire the complementary information from the two modalities to improve the classification performance.

This study had several limitations. First, the classical and well-established convolutional neural network, EffecientNet-b3, was selected as the backbone based on previous experiments, which may not be optimal. The characteristics of multimodal ultrasound imaging data need to be analyzed in-depth, and other deep neural networks will be attempted in the future to see if better performance can be achieved. Second, only images of tumor regions were cropped and used in data analyses, and regions of tumor periphery might be able to provide more information to improve model performance, which requires further experimental analyses. Third, our model was implemented using the ultrasound images collected from two hospitals only. A larger dataset acquired from more hospitals with different types or models of ultrasound equipment may have the potential to further improve the performance and generalization ability of our model.

## Data Availability

The original contributions presented in the study are included in the article/supplementary material, further inquiries can be directed to the corresponding authors.

## Conclusion

In this study, the proposed MUF-Net is able to classify benign and malignant solid renal tumors accurately, by extracting complementary features from B-mode and CEUS-mode images, which outperforms senior radiologists by a large margin.

## Abbreviations

AUC: area under the receiver operating characteristic curve; CEUS: contrast-enhanced ultrasound; CT: computed tomography; ccRCC: clear cell renal cell carcinoma; chRCC: chromophobe renal cell carcinomas; MRI: magnetic resonance imaging; MUF-Net: multimodal ultrasound fusion network; NPV: negative predictive value; PPV: positive predictive value; pRCC: papillary renal cell carcinomas; ROC: receiver operating characteristic.
